# Stem Cell-Based Therapies for Glaucoma Treatment: A Review Bridging the Gap in Veterinary Patients

**DOI:** 10.3390/ijms26010232

**Published:** 2024-12-30

**Authors:** Alícia de Sousa Moreira, Bruna Lopes, Ana Catarina Sousa, André Coelho, Patrícia Sousa, Ana Araújo, Esmeralda Delgado, Rui Alvites, Ana Colette Maurício

**Affiliations:** 1 Centro de Estudos de Ciência Animal (CECA), Instituto de Ciências, Tecnologias e Agroambiente (ICETA) da Universidade do Porto (UP), Praça Gomes Teixeira, Apartado 55142, 4051-401 Porto, Portugal; alicia.moreira.1998@gmail.com (A.d.S.M.); brunisabel95@gmail.com (B.L.); anacatarinasoaressousa@hotmail.com (A.C.S.); andrefmc17@gmail.com (A.C.); pfrfs_10@hotmail.com (P.S.); ruialvites@hotmail.com (R.A.); 2 Departamento de Clínicas Veterinárias, Instituto de Ciências Biomédicas de Abel Salazar (ICBAS), Universidade do Porto (UP), Rua de Jorge Viterbo Ferreira, n° 228, 4050-313 Porto, Portugal; araujoana_1@hotmail.com; 3 Associate Laboratory for Animal and Veterinary Sciences (AL4AnimalS), Faculdade de Medicina Veterinária (FMV), Universidade de Lisboa (UL), Avenida da Universidade Técnica, 1300-477 Lisboa, Portugal; esmeralda@fmv.ulisboa.pt; 4 Centro de Investigação Interdisciplinar em Sanidade Animal (CIISA), Faculdade de Medicina Veterinária (FMV), Universidade de Lisboa (UL), Avenida da Universidade Técnica, 1300-477 Lisboa, Portugal; 5 Instituto Universitário de Ciências da Saúde (CESPU), Avenida Central de Gandra n° 1317, 4585-116 Paredes, Portugal

**Keywords:** glaucoma, stem cell-based therapies, mesenchymal stem cells, secretome, umbilical cord mesenchymal stem cells, regenerative medicine, veterinary ophthalmology, veterinary medicine

## Abstract

Retinal diseases are characterized by progressive damage to retinal cells, leading to irreversible vision loss. Among these, glaucoma stands out as a multifactorial neurodegenerative disease involving elevated intraocular pressure, retinal ganglion cell apoptosis, and optic nerve damage, ultimately resulting in blindness in both humans and dogs. Stem cell-based therapies have emerged as a promising therapeutic option for such conditions due to their regenerative and neuroprotective potential. These therapies, particularly those based on mesenchymal stem cells, offer the potential to repair and protect retinal tissues through the bioactive molecules (growth factors, cytokines, chemokines) secreted, their secretome. However, research in this field, especially on the use of umbilical cord mesenchymal stem cells’ secretome, remains sparse. Most clinical trials focus on human glaucomatous patients, leaving a significant gap in veterinary patients’ application, especially in dogs, with additional research being needed to determine its usefulness in canine glaucoma treatment. Future studies should aim to evaluate these therapies across both human and veterinary contexts, broadening treatment possibilities for glaucoma.

## 1. Introduction

### 1.1. Description and Epidemiology

Retinal pathologies are a wide group of heterogenous degenerative and ischemic conditions that cause retinal cells apoptosis and loss of integrity. Within this diversity of diseases, the retinal degenerative ones (retinitis pigmentosa, diabetic retinopathy, age-related macular degeneration and glaucoma) stand out, since they are the main causes of irreversible vision loss and blindness worldwide, with a special emphasis on glaucoma in both humans and dogs [1,2,3,4,5]. Currently, glaucoma affects approximately 80 million people around the globe, and this number is expected to rise to more than 111 million by the year 2040 [2]. Likewise, around 1% of the dogs (2624 affected animals from a total of 294,314 purebred dogs included in the study) in North America and more than 50 different pure breeds are affected by this disease [5]. Unfortunately, despite the several therapeutic options available, success rates are very limited, especially in dogs, where in advanced-stage chronic cases enucleation is sometimes required to improve animals’ welfare and comfort, as this condition is often extremely painful [6].

Glaucoma is a progressive multifactorial neurodegenerative disease known in medicine since antiquity. Hippocrates defined “glaykoseis” as blindness that arises in older individuals, and Donders found that elevated intraocular pressure (IOP) caused blindness, naming the disease “Glaukoma simplex”, in 1862 [7]. The normal IOP values for dogs and cats usually vary between 15 mmHg and 25 mmHg [8]. Glaucoma is diagnosed when the IOP measured using a tonometer [9] exceeds 30 mmHg [10].

Previously, glaucoma was mainly defined by elevated IOP. However, it is now well established that while increased IOP is a major risk factor, other factors also play a significant role in the disease’s progression and the damage it causes, such as abnormalities in the extracellular matrix of the lamina cribrosa and defects in the blood flow to the optic nerve head (ONH). A notable example is normotensive glaucoma, which exhibits all the characteristics of glaucoma—such as retinal ganglion cell (RGC) dysfunction and apoptosis, optic nerve damage and degeneration, and progressive vision loss—despite normal IOP [11,12,13]. This form of glaucoma is acknowledged in both human [14,15] and non-human primates [16,17] and might also occur in dogs as well, although there is a lack of published data on this [12].

### 1.2. Pathophysiology

Concerning overall glaucoma pathophysiology, IOP increases due to inadequate drainage of the aqueous humor, which corresponds to the fluid in the anterior and posterior chambers produced by the ciliary body, located behind the iris. This fluid passes through the pupil and exits from the anterior chamber via the iridocorneal angle, which is positioned at the intersection between the cornea and the iris and includes the trabecular meshwork and Schlemm’s canal, this corresponding to the conventional outflow pathway. The unconventional route, or uveoscleral outflow, is estimated to carry 3–82% of total aqueous humor drainage across various species [18].

The production and drainage of aqueous humor occur nearly at the same rate, ensuring a constant IOP. Glaucoma develops because of inappropriate aqueous humor outflow, and the continuous high IOP promotes RGCs apoptosis, resulting in functional vision loss due to retinal degeneration [19]. Apoptosis of the RGCs, once initiated, is irreversible. The optic nerve head suffers structural and functional damage over time, including cupping of the optic disk, sectoral retinal nerve fiber layer and neuroretinal rim thinning, confirming the progressive course of the disease [19].

For many years, elevated IOP was believed to be the primary determinant in the pathogenesis of glaucoma, with therapeutic efforts focusing mainly its reduction and further maintenance. The latest research advances revealed that glaucoma is a multifactorial disease and, in human medicine, IOP is not reliable alone for diagnosing glaucoma, as many patients with this condition have normal IOP values [20].

Glaucoma arises from a complex interaction between multiple intertwined factors such as aging, genetic predisposition, abnormalities in aqueous humor dynamics, inflammation, and susceptibility to oxidative stress, all of which contribute to the progressive RGC degeneration and apoptosis and optic nerve atrophy [10,21].

Aging causes mitochondrial malfunction and decreased reactive oxygen species scavenging capability, making RGCs more vulnerable to damage. It also promotes oxidative stress (which damages cellular components), activates inflammatory pathways, accelerates apoptosis, and induces structural changes (such as lens stiffness) that obstruct aqueous outflow pathways, resulting in a vicious cycle of neurodegeneration [22,23,24].

Genetic predisposition is related to mutations affecting extracellular matrix, also leading to impaired outflow pathways and elevated IOP [25].

Abnormalities in aqueous humor dynamics, mainly at the trabecular meshwork and ciliary cleft, induce greater resistance to outflow due to extracellular matrix accumulation and fibrosis, worsening IOP rise [26].

Inflammatory processes disrupt the blood-aqueous barrier, activating cytokines such as Tumor Necrosis Factor Alpha (TNF-α) and triggering macrophage and glial cell responses that promote oxidative stress and tissue remodeling, further damaging the optic nerve [27,28].

The complexity of these mechanisms together emphasizes the need for a multifaceted therapeutic approach for the treatment of glaucoma.

### 1.3. Types of Glaucoma

Canine glaucoma can be either congenital, primary or secondary. Secondary glaucoma is quite common in veterinary medicine and occurs as a result of other ocular or systemic diseases.

**Figure 1:**
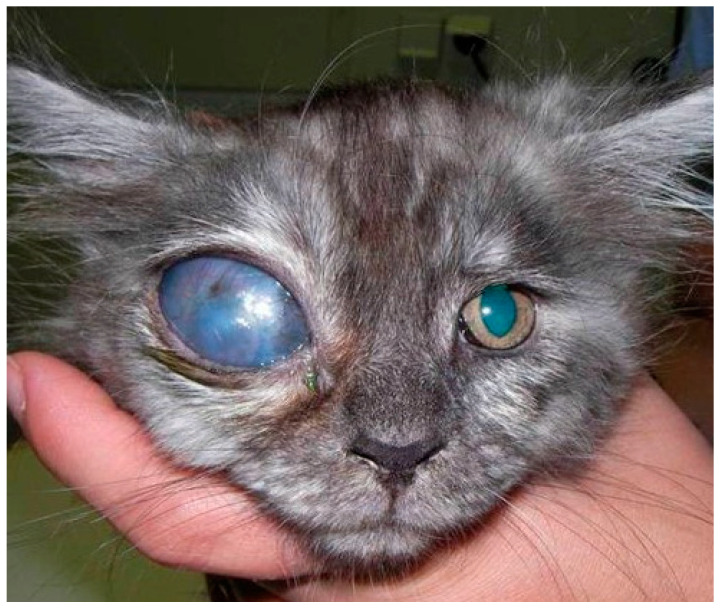
Congenital unilateral glaucoma in the right eye of a 3-month-old kitten with development of severe buphthalmia. The right ocular globe presents with severe enlargement accompanied by complete corneal edema and central corneal neopigmentation, impairing visualization of the anterior chamber and remaining intraocular structures. The left eye does not present with any signs of disease.

Congenital glaucoma is present at birth (Figure 1), being diagnosed in newborn animals through a complete and comprehensive ophthalmological exam, including gonioscopy on all littermates [10,29,30]. Typically, affected animals display abnormalities in the iridocorneal angle and pectinate ligament dysplasia. In these cases, treatment typically involves laser ciliary body ablation or enucleation, while evisceration and silicone prosthesis implantation can be an option when buphthalmos is mild and the fibrous tunica is preserved [8,10,30]. If the eyes are enucleated, the globes should be examined histologically to confirm the glaucoma’s cause.

**Figure 2:**
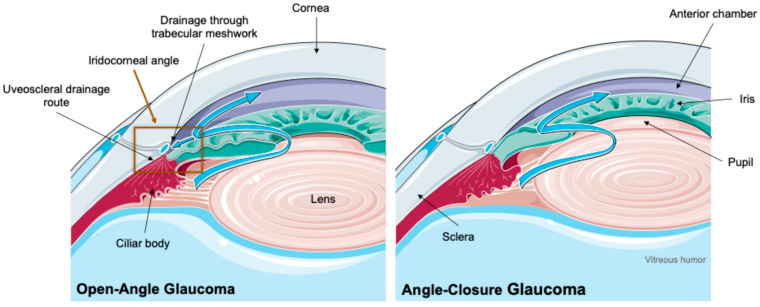
Comparison of open-angle and angle-closure glaucoma. In open-angle glaucoma, the drainage angle remains open, but the trabecular meshwork gradually becomes less efficient at allowing aqueous humor to exit, leading to increased intraocular pressure. In angle-closure glaucoma, the drainage angle is blocked, preventing the outflow of aqueous humor and causing a rapid rise in intraocular pressure. Images were used with permission from Servier Medical Art by Servier, licensed under a Creative Commons Attribution 4.0 International License.

Primary glaucoma, the most common presentation in dogs, can also be classified as primary open-angle glaucoma (POAG) or primary angle-closure glaucoma (PACG), with PACG being responsible for the majority of primary glaucoma cases (Figure 2) [10,31,32,33]. POAG can occur with both elevated and normal IOPs. When the IOP is considered normal (40% of POAG cases), it is referred to as a normotensive glaucoma [11,12,13].

Many breeds are predisposed to primary glaucoma in general, including the American Cocker Spaniel, Basset Hound, Beagle, Bouvier des Flandres, Dandie Dinmont Terrier, English Springer Spaniel, Irish Setter, Toy Poodle, Siberian Husky, Alaskan Malamute, Samoyed, Chow Chow, and American Eskimo [34].

POAG is characterized by a typical imbalance between the production and drainage of aqueous humor within the eye. Despite the drainage angle remaining open, impaired drainage leads to a gradual increase in the IOP. A study revealed that some dog breeds, such as the American Cocker Spaniel, Basset Hound, and Siberian Husky, are particularly predisposed to POAG [5].

On the other hand, PACG is characterized by a reduction in or closure of the drainage angle or a sudden blockage. This results in a compromised drainage of aqueous humor, causing a rapid increase in the IOP. The elevated pressure damages RGCs, paving the way for the onset of severe and painful eye conditions, including buphthalmia. Notably, PACG is the less common of the two primary glaucoma forms. Dog breeds such as English Springer Spaniel and Chow Chow, may be particularly predisposed to this form of the disease [5].

### 1.4. Prevalence and Risk Factors

Studies on the prevalence of glaucoma in dogs reveal significant variations across breeds and regions. Research from veterinary medical teaching institutions in North America, using data from the Veterinary Medical Database (VMDB) from 1964 to 2002, found substantial breed predispositions to primary glaucoma [5]. During the 38-year research period, the prevalence of primary glaucoma in pure-bred dogs climbed from 0.29% to 0.89% within selected breeds, such as the American Cocker Spaniel and Basset Hound, having notably high prevalence rates of 5.52% and 5.44%, respectively, by the end of the study [5]. These findings highlight the relevance of hereditary variables in the development of glaucoma, especially in breeds like the American Cocker Spaniel, Basset Hound, Boston Terrier, and Wire Fox Terrier, which were consistently amongst the most afflicted [5].

A study from the Veterinary College Hospital in Bangalore (2016–2019) found that glaucoma was responsible for 7.86% of the cases of ocular diseases in dogs, with the highest incidences in crossbred dogs (25%), Pomeranians (21.43%), and Labrador Retrievers (14.29%). Adult dogs (32.14%) and males (60.71%) were more frequently affected [35]. Recognizing these breed- and age-specific risks is crucial for early glaucoma detection, correct and appropriate diagnose and management, underscoring the need for targeted monitoring and potentially specialized therapeutic approaches across different regions or breeds.

Secondary glaucoma is a serious complication that can develop after intraocular disease such as uveitis, neoplasia, anterior lens luxation or following post-cataract surgery in dogs. In the last case the incidence of this condition has been reported to range from 5% to 19% within a two-year postoperative period [36,37,38]. However, in certain dog breeds, such as Boston Terriers, Shih Tzus, and Labrador Retrievers, the likelihood of developing secondary glaucoma after cataract surgery can increase significantly, reaching levels between 29% and 38% [36,37,38,39,40,41,42]. This elevated incidence in specific breeds points to the possibility of a hereditary component influencing the development of this condition. This suggests that genetic factors may play a significant role in the increased risk observed in these breeds.

**Figure 3:**
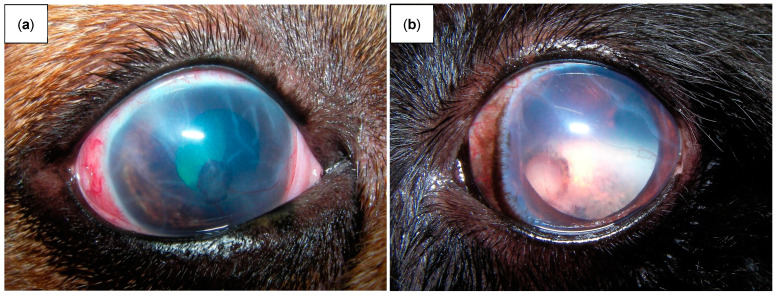
(**a**) Chronic glaucoma in a dog showing conjunctival and episcleral congestion, corneal edema, neovascularization and multiple corneal Haab’s striae corresponding to breaks in the Descemet membrane. You can also identify a ruptured iris cyst adherent to the corneal endothelium in the center of the cornea and a moderately dilated pupil where you can see a posterior subluxated lens. (**b**) Chronic glaucoma in a dog showing corneal edema, neopigmentation near the limbus, neovascularization and multiple corneal Haab’s striae. Through the completely dilated pupil, it is possible to see the fundus, showing marked retinal atrophy with complete retinal vascular attenuation, tapetal hyperreflectivity and optic disk atrophy and cupping.

Glaucoma symptoms vary depending on the stage of the illness, with acute and chronic phases exhibiting different symptoms. Early recognition of these symptoms, which vary from ocular discomfort, conjunctival and episcleral congestion and mydriasis in the acute phase to more severe structural alterations including optic nerve cupping, corneal Haabs striae and buphthalmia in the chronic phase (Figure 3), is critical for prompt management and preventing irreversible vision loss [29,32,43].

Human genetic testing in glaucoma has progressed substantially, shedding light on disease mechanisms, enhanced diagnose accuracy and patient management, and identification of potential therapeutic targets. Genes, such as myocilin (MYOC), optineurin (OPTN), and TANK-binding kinase 1 (TBK1), have been associated with early-onset glaucoma, while other genetic loci are associated with adult-onset types like POAG and PACG [44], although their use remains questionable [45]. The MYOC.mt1 variant’s role in POAG is still controversial, with mixed evidence on its impact [45]. Understanding the genetic keystones of glaucoma may facilitate the development of targeted therapies and improve the identification of individuals at higher risk [46].

### 1.5. Therapeutic Options (Medical and Surgical)

Current therapeutic interventions, including medical, physical, pharmacological, laser, and surgical procedures targeting IOP reduction, are limited and unsatisfactory. Despite their ability to delay the progression of the disease, none can effectively prevent vision loss and inevitable blindness, requiring the investigation of novel therapeutic possibilities, particularly those focusing on neuroprotection and neuroregeneration [47]. Emergency treatment used to start with the use of hyperosmotic agents like mannitol (1–2 g/kg IV over 20 min) to rapidly lower IOP. Since this effect is only temporary and the drug has potential side effects, this approach has been abandoned. It is critical to initiate longer-acting medications, such as carbonic anhydrase inhibitors and prostaglandin analogs, to lower the IOP [48]. Both topical and systemic carbonic anhydrase inhibitors (e.g., dorzolamide, brinzolamide, and methazolamide) decrease aqueous humor production, but systemic options like methazolamide may cause adverse side effects, such as, metabolic acidosis and gastrointestinal issues [49]. Topical agents, such as dorzolamide and brinzolamide, have fewer side effects.

Prostaglandin analogs are potent topical pharmaceuticals that should be used carefully in animals that have concomitant ocular disorders, such as severe uveitis or anterior lens luxation, since they induce miosis and exacerbate both conditions [50]. For long-term glaucoma management, maintaining low IOP is the crucial point to slower the progression of the disease. Prostaglandin analogs, such as latanoprost, aid to increase uveoscleral outflow by activating prostaglandin F receptors, inducing miosis, leading to the relaxation of the ciliary body muscle and enhancing fluid drainage [49].

In human medicine, beta-adrenergic antagonists like 0.5% timolol maleate are commonly used but are generally less effective in veterinary patients due to their mild impact on lowering IOP [49]. Considering this and the fact that medical management offers only temporary relief, surgical intervention might be necessary, both in animals and in humans [49]. Among the last U.S. Food and Drug Administration (FDA)-approved medications for human glaucoma treatment (2017–2023) are Latanoprostene Bunod (Vyzulta™, Bausch & Lomb Incorporated, Tampa, FL, USA) and Netarsudil (Rhopressa™, Aerie Pharmaceuticals, Inc., Durham, NC, USA) [51], approved in November and December of 2017, respectively. Latanoprostene bunod, a nitric oxide-donating prostaglandin F2α agonist, has shown superior IOP-lowering effects compared to latanoprost, particularly in specific canine models of POAG [52,53,54,55]. This medication can improve uveoscleral and trabecular outflow pathways [52,53,54,55]. Netarsudil, a Rho kinase (ROCK) inhibitor, targets the trabecular meshwork cells, reducing cell stiffness and contractility, and lowering episcleral venous pressure, thereby increasing the outflow capacity and lowering IOP [56,57,58,59,60]. Although its effectiveness in dogs is still being studied, netarsudil appears promising when used in combination with latanoprost for improved control of IOP, as observed in human trials [56,57,58,59,60,61]. In March 2019, FDA approved Rocklatan™ (Aerie Pharmaceuticals, Inc., Durham, North Carolina, USA) [62], a fixed-dose combination of netarsudil and latanoprost that enhances IOP lowering by combining both the ROCK inhibition pathway and the uveoscleral outflow pathway. One year later, Durysta™ (Allergan, Inc., Irvine, California, USA) [63] was approved, an implant containing bimatoprost that slowly releases the medication over time to lower IOP. This implant is designed for patients with POAG and provides an alternative to daily eye drops. Approved in September 2022, Omlonti™ (Omidenepag Isopropyl, Santen Pharmaceutical Co., Ltd., Osaka, Japan) [64] works as a receptor agonist of prostaglandin EP2, increasing aqueous humor drainage. This drug was also designed for the treatment of POAG, specifically. Finally, FDA approved iDose^®^ TR [65] in 2023, a travoprost intracameral implant delivering continuous medication directly into the eye for up to three years.

Researchers are developing innovative approaches such as preservative-free formulations, improved delivery methods, and new formulations to boost patient adherence and address common treatment barriers [66]. Erythropoietin (EPO), known for its anti-apoptotic, antioxidant, and anti-inflammatory effects, has shown promise for neuroprotection in glaucoma [67]. This hormone is also being tested, through different formulations, in animal pre-clinical trials to access its efficacy for the treatment of veterinary glaucomatous patients [68,69,70,71,72]. Additionally, bis(7)-tacrine, nimodipine, and mirtogenol are being considered as adjunct therapies to counteract oxidative stress, vascular dysfunction, and retinal cell apoptosis [73]. Memantine, an N-methyl-D-aspartate receptor blocker, also holds potential as a neuroprotectant for glaucoma, with studies noting a favorable safety profile [74]. Together, these emerging therapies may offer more effective glaucoma management and reduce the disease’s progression-related morbidity and economic impact [74].

Contrary to human medicine, medical treatment options in dogs are not very effective. Moreover, one of the main challenges of canine glaucoma treatment is ensuring that the prescribed treatment (usually, eye drops) regimen is followed consistently and correctly by the owners. Non-compliance can greatly reduce the effectiveness of glaucoma management and is one of the reasons for treatment failure. To overcome this issue, sustained-release drug delivery systems, that offer the potential to provide more consistent therapeutic outcomes and reduce the frequency of eye drop administration, are being investigated [75,76,77,78,79,80,81,82,83,84]. Unfortunately, these procedures are not yet widely studied and adopted in veterinary medicine.

As previously mentioned, in human medicine, medical treatment is usually effective. On the contrary, in dogs, medical treatment alone is not sufficient and surgical options are necessary. These procedures aim at two targets: to enhance aqueous humor outflow [85], such as anterior chamber gonioimplants, and reduce its production through laser photocoagulation, endolaser or cyclocryotherapy [86]. In severe nonresponsive cases of glaucoma leading to blind painful eyes, the treatment involves evisceration and intrascleral prosthesis implantation or enucleation [87].

Secondary glaucoma is treated similarly, with the goal of restoring or preserving aqueous humor outflow, which might be blocked at the iridocorneal angle or at the pupil. The etiology may differ, making diagnosis and therapy more challenging [87]. In these cases, it is important to identify and address the cause of the glaucoma.

Recent medical treatments for glaucoma, designed primarily for the human eye, may prove ineffective when applied to dogs’ eyes. It underscores the significance of comprehending the anatomical and physiological characteristics of the aqueous humor outflow pathways in both species. Glaucoma in dogs is similar to humans in terms of RGC loss and optic nerve neuropathy, although the aqueous humor outflow pathways differ between these species. For instance, conditions like pectinate ligament dysplasia, which is recognized as a significant risk factor for the development of POAG in dogs, do not hold the same relevance in humans [88]. Another important distinction between the human and the dog eyes relies in the post-trabecular meshwork outflow pathways, specifically the human Schlemm’s canal compared to the canine angular aqueous plexus. While the human Schlemm’s canal has been extensively studied, there is still much to be discovered about the function of the canine angular aqueous plexus and its role in the development of glaucoma [88]. Moreover, there is a need to expand our understanding of the risk factors and mechanisms of canine glaucoma, including the differences between dogs and other species, and even between each dog breed. This highlights the species-specific factors in glaucoma pathogenesis, underscoring the importance of targeted research for better understanding and treating glaucoma in canine patients. In summary, stem cell-based therapies are still in their experimental stages for dogs, and there is an urgent need for more robust and diverse veterinary studies. The available treatments and clinical trials are designed for humans mainly, with limited or no adaptations for veterinary use, despite the similarities in the progression of the disease across species [14,88,89]. This inconsistency leaves a significant gap in understanding how stem cell therapies can be optimized for dogs, where glaucoma is equally painful and progressive leading to irreversible blindness.

Another critical factor influencing treatment effectiveness includes diagnostic tools, disease recognition and staging. Early detection and precise diagnosis are closely linked to heightened therapeutic efficacy in preventing visual loss [88].

Given the limitations of current treatment options for canine glaucoma, such as the temporary relief (IOP-lowering mechanisms) or the invasive procedures, there is a growing need for novel therapeutic strategies.

Regenerative medicine and cell-based therapies are emerging as a promising solution, with the use of stem cells’ and their biofactors as a potential tool to regenerate damaged ocular tissues and address the root causes of glaucoma, ideally avoiding vision impairment. This approach not only offers hope for more effective long-term management but also represents a significant advancement in veterinary medicine ophthalmology.

## 2. Stem Cells Basics (Types, Sources, Advantages and Limitations)

Cell-based therapies have emerged as a prominent area of focus within regenerative medicine research. Stem cells can be classified according to their source as embryonic, adult (hematopoietic stem cells (HSCs) and non-hematopoietic/mesenchymal stem/stromal cells (MSCs)) or induced pluripotent stem cells (iPSCs) [90,91,92,93], and according to their stage of development and differentiation as totipotent, pluripotent or multipotent cells [92,93,94].

MSCs can be defined as adult multipotent cells with a mesodermal origin that are able to replicate as undifferentiated cells, differentiate into various cell lineages (related to adipose, tendon, muscle, cartilage, bone, skin and connective tissues) and self-renew [34,92,93,95,96,97,98]. These characteristics, the ease and success of harvesting, along with few ethical restraints and the potential therapeutic application of these stem cells, have given them wide popularity in many clinical and laboratory investigation areas.

The appeal of MSCs lies not only in their differentiation abilities but also in their interaction with the immune system. MSCs can modulate immune responses by secreting a range of bioactive molecules, collectively known as the secretome, which includes cytokines, growth factors, and chemokines. The secretome can prevent apoptosis [99] and reduce fibrosis [100], promote wound healing, support proliferation and angiogenesis and the differentiation of cells in situ [101]. It can also reduce the inflammatory response and, consequently, enhance MSCs’ maintenance and the regeneration environment [102], making these cells highly valuable in therapeutic applications.

As previously mentioned, stem cells’ therapeutic effect is, currently, essentially attributed to their secretome. Studies have been conducted to understand if it is crucial or not to apply stem cells in injured tissues, once their secretome can lead to similar regenerative outcomes [103,104]. MSCs’ culture supernatant, obtained in vitro after subjecting cells to specific culture conditions, rich in specific bioactive factors secreted to the extracellular space (such as cytokines and growth factors), is known as Conditioned Media (CM). CM’s composition fluctuates according to cells’ stimulation, stem cells type and tissue [105]. Using CM, instead of stem cells in regenerative treatments, diminishes the risk of applying cells with malignant characteristics, as well as the risk of rejection. Similarly, cell dosage and storage requirements may be reduced with an increased response capacity in emergency/acute situations [106].

In veterinary medicine, MSCs have been sourced from various tissues in cats, dogs, and horses, such as the umbilical cord stroma and blood [107], dental pulp and periodontal ligament [108], bone marrow [109], adipose tissue [109], synovial membrane and fluid [110] and peripheral blood [111]. Remarkably, MSCs were isolated from mice bone-marrow [112] for the first time ever and their first application in veterinary medicine history was performed in a horse with suspensory ligament desmitis [113].

Bone marrow and adipose tissue are the most common sources of MSCs for therapeutic purposes, even though MSCs can be virtually isolated from any body tissue niches or organ [114]. This is primarily since these sources yield a higher number of cells for collection and isolation. However, harvesting, for example, bone marrow mesenchymal stem cells (BM-MSCs) is an invasive and painful process. Additionally, studies have shown that due to aging, there is a decline in the therapeutic potential of BM-MSCs and adipose-derived MSCs (AD-MSCs) [115]. Given these considerations, the umbilical cord is increasingly recognized as an outstanding alternative source of MSCs, gaining significant attraction in regenerative medicine. The collection of umbilical cord tissue immediately after a cesarean section is a straightforward, noninvasive procedure that circumvents the ethical issues associated with bone marrow extraction. Historically, the umbilical cord was discarded as medical waste, underscoring the fact that its use in stem cell therapies has no adverse effects on either the mother or the newborn. Moreover, the umbilical cord tissue is an excellent reservoir of young stem cells (less mature when compared to other MSCs’ sources), with high proliferative, growth and differentiation potential [116,117]. Umbilical cord mesenchymal stem cells (UC-MSCs) are also known for long-term viability at higher passages and for having low immunogenicity [117]. As a result, UC-MSCs are well tolerated in allogeneic cell therapies (the use of stem cells obtained from a donor who is different from the recipient, but from the same species) [118]. Another great advantage of UC-MSCs is that they are non-tumorigenic [119].

Conversely, obtaining umbilical cord tissue or matrix in veterinary medicine can be challenging. Cesarean sections are infrequently performed, typically only when medically necessary, such as in cases of dystocia. Additionally, many pet owners today prioritize sterilizing their animals, further limiting opportunities to collect this material [120]. Moreover, during natural births, the mother’s maternal instinct often results in the ingestion of birth materials, including the umbilical cord. In other species, like horses, it is not practical to collect this tissue in a sterile manner because births occur in the field. Despite these challenges, the availability of stem cell banking options, both public and private, has increased globally over time [121], making it easier to access canine umbilical cord mesenchymal stem cells (cUC-MSCs) and enabling a quicker response to therapeutic needs.

So far, in veterinary medicine, MSCs have been principally studied and used in horses and dogs, for the treatment of orthopedic disorders [122]. Nevertheless, encouraging results prompted the pursuit of new studies about MSCs’ therapeutic potential application in other diseases (ocular, liver, renal, dermal, neuronal, olfactory, respiratory, reproductive, and digestive diseases) [123].

The Mesenchymal and Tissue Stem Cell Committee of the International Society for Cellular Therapy (ISCT) established three minimal criteria to standardize human MSCs characterization. Briefly, human MSCs can be defined by their capacity to adhere to plastic surfaces and their fibroblastic-like morphology when cultured in flasks; their ability to differentiate into at least three different cell lineages (chondrogenic, osteogenic and adipogenic) when cultured in appropriate differentiation culture conditions; and expression of clusters of differentiation (CDs) like CD105, CD73, and CD90 and reduced/non-expression of hematopoietic markers (CD45, CD34, CD14 or CD11b, CD79α or CD19), and major histocompatibility complex- (MHC-) II/human leukocyte antigen- (HLA-) DR [97].

Unfortunately, to date, no standardized guidelines have been approved for characterizing veterinary MSCs and their bioproducts, as the antibodies commonly used are specific to human stem cells. As a result, human guidelines remain the only reference available. Even though human MSCs-based therapies have been studied deeply, and uniform factors were established to consider them as effective and safe [124], the diversity of veterinary species, their biological differences, and MSCs’ heterogeneous phenotypes and behavior in vitro make it difficult to describe specific patterns for the therapeutic use of MSCs and their safety in veterinary medicine. Current research focuses on finding new critical surface markers, such as CD271 and STRO-1, with the aim of establishing standardized and reliable guidelines for veterinary medicine MSC characterization [125,126].

MSCs found in fetal tissues, placenta, umbilical cord, and various adult tissues such as bone marrow and adipose tissue [127,128] offer additional advantages: they secrete specific bioactive molecules of interest, such as growth factors (Epidermal Growth Factor (EGF), Vascular Endothelial Growth Factor A (VEGF-A), Fibroblast Growth Factor (FGF)), cytokines (TNF-α, Interleukin-6 (IL-6), Interleukin-8 (IL-8)), and other survival-promoting agents like insulin growth factor 1 (IGF-1) and transforming growth factor (TGF)-β1; they express homing receptors to migrate to damaged or inflamed areas [129,130]. A limitation of MSCs is their short lifespan post-transplantation, which may reduce their effectiveness, and once differentiated, they can increase immunogenicity by expressing major histocompatibility complex I (MHC-I) and MHC-II molecules. In systemic transplantation, many MSCs are also trapped in the lungs, increasing the risk of pulmonary thrombi, which may further limit their therapeutic potential. It is also known that MSCs tend to be attracted to places where some level of inflammation is occurring, which can lead to non-specific systemic dispersion and reduce the load of cells that reach the place where the therapeutic action is expected. Compared to bone marrow and adipose tissue stem cells, or even umbilical cord blood stem cells, UC-MSCs represent a promising therapeutic option in veterinary regenerative medicine, though they have received relatively little research attention to date.

Considering glaucoma therapy, stem cells have numerous beneficial characteristics: (1) their capacity to differentiate into different cell types, allowing for the selective replacement of RGCs; (2) their neuroprotective and immunomodulatory properties, particularly MSCs [131]; (3) their low immunogenicity, especially in pluripotent stem cells; (4) the secretome, which aids in injury repair and immunomodulation, often achieving therapeutic benefits without the need for full integration into the host tissue [132]; (5) their potential to serve as delivery systems for neurotrophic and growth factors and anti-apoptotic factors [131].

Preclinical studies using pluripotent stem cells, such as embryonic stem cells (ESCs) and iPSCs, have paved the way for their consideration in clinical applications for glaucoma. Pluripotent stem cells are particularly advantageous since they remain undifferentiated for extended periods in culture and can differentiate into every somatic cell type. Takahashi et al. reported iPSCs in 2007 [133], which are generated from adult somatic tissues via genetic engineering, having the potential to prevent immune rejection and reduce the requirement for immunosuppressive drugs. Nevertheless, ESCs and iPSCs oppose obstacles such as genetic instability and the potential of developing teratomas [134], and iPSCs need more work to grow properly rather than adult stem cells.

## 3. Stem Cells in Glaucoma Treatment

The neuroprotective and differentiation capabilities of MSCs place them as a potential therapeutic strategy for glaucoma treatment. Research has shown that MSCs can support RGC survival and resistance and may even differentiate into these cells, integrating into the retina [135,136], by releasing growth factors, cytokines, and chemokines—collectively known as the secretome—regulating the cellular environment, and mitigating tissue damage caused by excessive immune responses [137,138]. Likewise, other studies have proven that BM-MSCs could integrate into retinal tissue after intravitreal injection and last for several weeks [139,140,141,142]. This provided significant IOP reduction and was related to neurotrophic factor secretion, as Brain-Derived Neurotrophic Factor (BDNF) and Ciliary Neurotrophic Factor (CNTF), which may inhibit RGC and photoreceptor apoptosis, maintain functional vision and avoiding progressive blindness [139,140,141,142].

Along with laser-induced regeneration, MSC therapies in glaucoma models also exhibit temporary increases in RGC numbers and axons, promoting functional recovery, as demonstrated by enhanced performance on visual-guided tasks [143,144,145]. Moreover, a study conducted by Jiang et al. in 2019 showed that iPSC-derived MSCs had the capacity to transfer their mitochondria to the RGCs of mice, promoting cell survival and lowering in situ inflammation after injection [138].

In advanced stages of glaucoma, the only potential possibility to restore vision or at least impede the progression of the disease lies in the regeneration of the optic nerve, making stem cell-based therapies a promising yet very challenging approach, with numerous related difficulties.

One of the first challenges is identifying a reliable source of stem cells capable of differentiating into RGCs. Once the appropriate stem cells are selected, these cells must be transplanted into the eye and integrated into the retina, and this process requires a permissive environment for axonal regrowth. Unfortunately, in the central nervous system (CNS), glial scarring—formed in response to injury—acts as a significant barrier to neural repair [146]. In glaucoma, this problem is exacerbated by the altered biochemical microenvironment, where activated astrocytes in the lamina cribrosa and Müller cells in the retina hinder axon regeneration [146].

Addressing these environmental and cellular changes is critical to promoting successful axon growth through the ONH. Beyond the mechanical challenges, the transplanted cells must also establish functional synaptic connections with retinal interneurons and corresponding neurons in the brain. Only through this precise integration can the visual function be preserved or restored.

Despite the theoretical potential of stem cell-based therapies, translating success from preclinical studies in animal models to human clinical applications has proven difficult. Most animal studies are conducted using induced models of glaucoma, where the damage is acute and less extensive compared to the chronic, progressive nature of glaucoma [147]. In these models, the therapeutic environment may be more favorable, allowing for better outcomes than what can be realistically achieved in humans, where optic nerve degeneration often takes years to develop [147]. Moreover, as previously mentioned, biological differences between animal models and humans, such as glial scarring and the cellular microenvironment, further complicate this translation [148].

One of the significant challenges in addressing veterinary patients’ glaucoma, especially dogs, is the absence of preclinical studies focused on animals. While there has been distinguished progress in the application of stem cell-based therapies for treating human ocular conditions, the application of these therapies for glaucoma remains underexplored in animal models, particularly in canine patients. The majority of stem cell-based therapies in canine ophthalmology have been focused on treating corneal ulcers [149,150,151] and keratoconjunctivitis sicca [152,153,154,155,156,157]. Nevertheless, there are no clinical studies considering the potential of these therapies for canine glaucoma [158,159].

As mentioned above, human clinical trials for glaucoma, although limited, are more prevalent. These trials often involve small participant groups and have not yet demonstrated long-term efficacy or significant benefits [160,161]. Severe complications, including retinal detachment and proliferative vitreoretinopathy, have also been reported in human trials [162], which further highlights the limitations and risks associated with these emerging treatments.

The following table (Table 1) summarizes clinical studies registered on various platforms, including the WHO International Clinical Trials Registry Platform (ICTRP), ClinicalTrials.gov, and the European Clinical Trials Register, with an emphasis on the use of stem cells for glaucoma treatment. These studies investigate the ability of stem cell therapies to regenerate ocular tissues, preserve the optic nerve, and restore vision due to glaucoma. Studies are in diverse phases, ranging from early preclinical trials evaluating safety and efficacy in animal models to human clinical trials looking into therapeutic results. This table summarizes the current global research activities in this novel field of glaucoma therapy.

Regrettably, there are only a few registered clinical trials, and many have not yet reported results. Most trials involve small patient samples, with the exception of SCOT1 and SCOT2, which focused on using autologous BM-MSCs for different retinal and optic nerve diseases where the inclusion of glaucoma patients in these studies has been relatively limited. NCT06200727 also includes a significant patient population; however, the number of glaucoma patients enrolled in this trial remains relatively low.

The clinical trial NCT06498440 provides the most extensive results concerning specifically glaucoma, though its’s focused on the effectiveness of steroid and non-steroid anti-inflammatory drugs, highlighting the need for more research and investigation of the potential of cell-based therapies. Among the other trials, NCT02330978 is the only one that has produced outcomes on the use of MSCs for glaucoma treatment. Results showed no improvement in visual acuity or field [177]. One patient developed retinal detachment and was removed, while the other maintained stable retinal function without significant changes during the study [177].

Despite the limited number of animal studies using stem cell-based therapies for glaucoma treatment, Roubeix et al. (2015) conducted a study to assess the potential of BM-MSCs in a rat model of ocular hypertension. In this study, cells were administered into the anterior chamber of 20 hypertensive Long–Evans rats [181]. The results revealed a significant and long-lasting reduction in the IOP and meaningful neuroprotective effects on RGCs [181]. These findings highlight the potential of MSCs for the treatment of glaucoma in veterinary patients and the need for further studies on this matter.

## 4. Conclusions

Glaucoma has a substantial influence on dogs’ quality of life, being an extremely painful condition and causing gradual visual loss with inevitably general deterioration in well-being. Despite its ubiquity, long-term therapeutic options are limited, especially due to owners’ compliance, highlighting the need for novel treatments. Stem cell-based therapies demonstrate potential in the management of this condition. Current research into the use of stem cells for the treatment of glaucoma, particularly umbilical cord mesenchymal stem cells and their secretome, is limited. The bulk of research has been undertaken on humans or animal models to evaluate potential human therapy, with relatively little conducted on veterinary patients such as dogs. Given the intriguing potential of these medicines, more extensive studies should be conducted in the future, particularly to investigate their use in veterinary medicine, where glaucoma treatment options remain scarce.

## Figures and Tables

**Table 1:** Overview of clinical trials investigating stem cell-based therapies and other therapeutic options for glaucoma treatment.

Study Title	NCT Number, Location and Date	Eye Conditions	Treatment	Status	Sample Size/Target	Results
Safety of Cultured Allogeneic Adult Umbilical Cord Derived Mesenchymal Stem Cells for Eye Diseases	NCT05147701 Antigua, Barbuda, Argentina (2022–2026)	Macular degeneration optic atrophy glaucoma etc.	AlloRx (100 million cultured allogeneic adult UC-MSCs, intravenous and sub-tenon delivery)	Recruiting	20	[163,164,165]
Stem Cell Ophthalmology Treatment Study II (SCOTS2)	NCT03011541 United States, United Arab Emirates (2016–2026)	Age-related macular degeneration glaucoma etc.	Arm 1 (Autologous BM-MSCs provided retrobulbar, sub-tenon and intravenous)	Recruiting	500	[166,167,168,169,170,171,172,173,174,175,176]
Intravitreal Mesenchymal Stem Cell Transplantation in Advanced Glaucoma	NCT02330978 Brazil (2014–2019)	Retinal degeneration POAG	Intravitreal transplantation of autologous BM-MSCs	Completed	2	[177]
Stem Cell Ophthalmology Treatment Study (SCOTS)	NCT01920867 United States, United Arab Emirates (2013–2019)	Optic nerve disease glaucoma etc.	Intraocular, retrobulbar, intravenous, sub-tenon, intravitreal transplantation of autologous BM-MSCs	Unknown	300	[166,167,168,169,178]
Platelet-rich Fibrin (PRF) Membrane in Ophthalmic Diseases	NCT06200727 China (2023–2025)	Platelet-rich fibrin macular holes pterygium glaucoma	Autologous PRF membrane grafting/amniotic membrane to cover the exposed sclera after trabeculectomy for glaucoma	Active, not recruiting	170	[179,180]
Effectiveness and Safety of Adipose-Derived Regenerative Cells for Treatment of Glaucomatous Neurodegeneration	NCT02144103 Moscow, Russian federations (2014–2019)	Retinal degeneration POAG	Liposuction to isolate and concentrate AD-MSCs. The concentrate is then injected into the sub-tenon space of the patient’s eye.	Unknown	16	No results reported yet
Efficacy of NSAID vs. Steroid-NSAID Combo Post-Selective Laser Trabeculoplasty: Phase 4, Single-Center RCT (CES-NSLT)	NCT06498440 Canada (2024–2025)	Open-angle glaucoma ocular hypertension etc.	Ketorolac 0.5% eye drops; Ketorolac 0.5% and Fluorometholone 0.1% eye drops	Not yet recruiting	126	No results reported yet

## Data Availability

Not applicable.

## Abbreviations

AD-MSCs	Adipose tissue mesenchymal stem cells
BDNF	Brain-Derived Neurotrophic Factor
BM-MSCs	Bone marrow mesenchymal stem cells
CDs	Clusters of differentiation
CM	Conditioned Media
CNS	Central nervous system
CNTF	Ciliary Neurotrophic Factor
cUC-MSCs	Canine Umbilical Cord Mesenchymal Stem Cells
EGF	Epidermal Growth Factor
ESCs	Embryonic stem cells
FDA	Food and Drug Administration
FGF	Fibroblast Growth Factor
HLA	Human Leukocyte Antigen
HSCs	Hematopoietic stem cells
ICTRP	International Clinical Trials Registry Platform
IGF-1	Insulin-like Growth Factor 1
IL-6/-8	Interleukin-6/8
IOP	Intraocular pressure
iPSCs	Induced pluripotent stem cells
ISCT	International Society for Cellular Therapy
MHC-I/-II	Major histocompatibility complex I/II
MSCs	Mesenchymal stem cells
MYOC	Myocilin
ONH	Optic nerve head
OPTN	Optineurin
PACG	Primary angle-closure glaucoma
POAG	Primary open-angle glaucoma
PRF	Platelet-rich fibrin
RGCs	Retinal ganglion cells
TBK1	TANK-binding kinase 1
TGF-β1	Transforming Growth Factor-beta 1
TNF-α	Tumor Necrosis Factor Alpha
UC-MSCs	Umbilical cord mesenchymal stem cells
VEGF-A	Vascular Endothelial Growth Factor A
VMDB	Veterinary Medical Database
